# Optimization of topological complexity for one-dimensional arterial blood flow models

**DOI:** 10.1098/rsif.2018.0546

**Published:** 2018-12-12

**Authors:** Fredrik E. Fossan, Jorge Mariscal-Harana, Jordi Alastruey, Leif R. Hellevik

**Affiliations:** 1 Norwegian University of Science and Technology, Trondheim, Norway; 2 Department of Biomedical Engineering, King’s College, London, UK; 3 Institute of Personalized Medicine, Sechenov University, Moscow, Russia

**Keywords:** one-dimensional blood flow, model reduction, model optimization, haemodynamics, pulse wave analysis, computational instantaneous wave-free ratio

## Abstract

As computational models of the cardiovascular system are applied in modern personalized medicine, maximizing certainty of model input becomes crucial. A model with a high number of arterial segments results in a more realistic description of the system, but also requires a high number of parameters with associated uncertainties. In this paper, we present a method to optimize/reduce the number of arterial segments included in one-dimensional blood flow models, while preserving key features of flow and pressure waveforms. We quantify the preservation of key flow features for the optimal network with respect to the baseline networks (a 96-artery and a patient-specific coronary network) by various metrics and quantities like average relative error, pulse pressure and augmentation pressure. Furthermore, various physiological and pathological states are considered. For the aortic root and larger systemic artery pressure waveforms a network with minimal description of lower and upper limb arteries and no cerebral arteries, sufficiently captures important features such as pressure augmentation and pulse pressure. Discrepancies in carotid and middle cerebral artery flow waveforms that are introduced by describing the arterial system in a minimalistic manner are small compared with errors related to uncertainties in blood flow measurements obtained by ultrasound.

## 1. Introduction

Computational models of the cardiovascular system are commonly separated into three-dimensional (3D), one-dimensional (1D) and lumped models (0D). One of the first attempts to model pressure and flow waveforms was through the classical 0D Windkessel (WK) model [1]. A noteworthy extension to this was presented in [2] where a resistance element representing the characteristic impedance was added, and many variations and extensions have been proposed [3]. The most important drawback of the family of 0D models is inherent in the assumption of infinite wave velocity and that spatially distributed parameters are modelled as single point parameters.

Through the years distributed models with various degrees of detail have been suggested. In [4,5], the systemic circulation was modelled as two asymmetric parallel branches, one supplying the head and upper limbs, and one supplying the rest of the body. In [6], a model consisting of the 33 largest systemic arteries was tested using an *in vitro* experiment. In [7], the arterial network was expanded to include 55 arterial 1D segments. In [8], a complete description of the systemic arterial tree containing the largest arteries of the head and upper and lower body was validated using *in vivo* measurements. The study also includes a detailed overview of 1D models up until 2009, highlighting their variation in detail and complexity. More recently, in [9], a model accounting for pulse wave propagation in all regions of the circulation including approximately 400 arteries and 350 veins was presented. Yet others have modelled the arterial system in a very high level of detail including more than 2000 arteries [10,11].

We have come a long way in creating realistic and detailed descriptions of the entire arterial tree and circulatory system. However, given the near endless number of small arteries and capillaries in the human body, the network has to be truncated at a certain level. Since reliable measurements of flow or pressure at all terminal sites are practically impossible to obtain, outflow boundary conditions are commonly set through simpler models representing the peripheral circulation. Indeed the above-mentioned family of 0D WK models have been the preferred choice for providing boundary conditions at terminal branches.

There is little consensus in the scientific community on the level of detail of the computational domain. Furthermore, few studies have focused on the errors and limitations associated with truncating the arterial network at given sites. In [8], they state that a detailed description of the cerebral circulation is required in order to attain accurate and physiological flow predictions in the common carotid artery. In [12], they found that the arterial tree could be truncated after the first generation of bifurcations without significantly altering pressure and flow waveforms, if matched three-element WK outflow models were used. In [13], they presented a method for lumping 1D arterial segments into three-element WK models and applied their method on a network of 55 arteries (excluding the circle of Willis).

**Figure 1.**
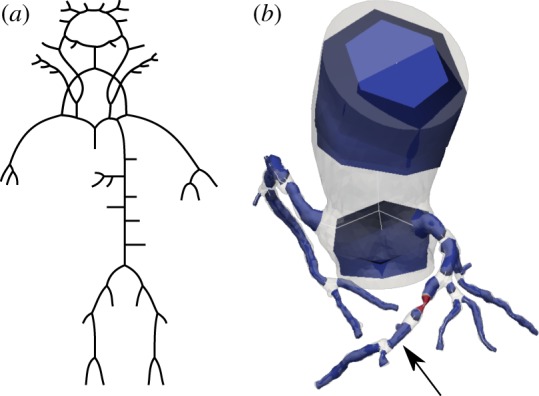
Two baseline models were used in this work: a model containing 96 arterial segments in which parameters and topology were adapted from [9] (*a*), and a patient-specific coronary network (*b*). The arrow indicates the location of invasive pressure measurements, and the section coloured in red is a significant stenosis.

Here, we present a sound mathematical framework that enables us to find the necessary arteries to include for a given clinical application. The framework involves finding the model with the fewest number of arteries that is still able to produce pressure and flow waveforms below a certain error threshold compared with a corresponding detailed (baseline) model (figure 1). This approach reduces the number of uncertain input parameters, while still assuring that the simplifications do not limit the model predictions. We illustrate the framework for different clinically relevant quantities of interest: central aortic and larger systemic artery pressure waveforms, common carotid and middle cerebral artery flow waveforms and coronary pressure waveforms. We note that our framework is intended to be used in an early stage as a tool for model selection that aims at minimizing total uncertainty.

## 2. Material and methods

### 2.1. Framework for balancing topological complexity with model error

Here, we present a framework for reducing the number of vessel segments still assuring wanted features of pressure and/or flow to be within acceptable agreement with the corresponding full model:

— Define a baseline model.
— Locate the quantity of interest appropriate for the problem (e.g. aortic pressure and/or carotid flow).
— Define a threshold for pressure and/or flow (e.g. RMS-error, pulse or mean pressure).
— Create reduced models by applying the methods described in §2.4.1 or §2.4.2, and solve the 1D networks.
— Find the network with the fewest number of arteries subject to the constraint of the threshold.

### 2.2. Arterial baseline models

We applied our new methodology on two arterial models, both illustrated in figure 1.

#### 2.2.1. Systemic arterial network

The first baseline model considered includes 96 of the largest systemic arteries, in which parameters and geometry were adapted from Mynard *et al.* [9]. They compared model-derived pressure/flow waveforms with published *in vivo* waveforms from healthy adults, validating the model’s capability of providing realistic waveforms throughout the arterial tree.

#### 2.2.2. Coronary network

The second baseline model considered in this work was based on a series of invasive and non-invasive measurements of a patient (sex: female, age: 58, height: 162 cm, weight: 78 kg) with positive findings of stable coronary artery disease after clinical inspection and coronary computed tomography angiography (CCTA) examination. The data were collected as part of an ongoing clinical trial at St Olavs Hospital, Trondheim, Norway [14]. Cardiac output (CO) was measured by transthoracic Doppler echocardiography using a GE Vivid E95 scanner (GE Vingmed Ultrasound, Horten, Norway). The patient was further referred to invasive angiography, and a Verrata Plus (Philips Volcano, San Diego, USA) pressure wire was used to obtain pressure tracings at the coronary ostium and distal of an epicardial stenosis. Proximal, *P*_p_ and distal, *P*_d_ pressure tracings are shown in figure 8. The last 30% of the cardiac cycle is highlighted and was used to compute the instantaneous wave-free ratio (iFR), which is a drug-free index of the significance of the stenosis [15]. Measurement of fractional flow reserve (FFR) [15], obtained during drug-induced hyperaemia (maximum coronary flow) was also available. The coronary geometry was segmented using the open-source software ITK-SNAP [16], the surface was then meshed using the open-source library Vascular Modeling ToolKit [17]. 1D domains were extracted from the 3D volume mesh by computing equivalent axisymetric cross-sectional areas along centrelines. Stenotic regions were automatically detected using a Gaussian filter-based approach [18].

### 2.3. Numerical formulation

#### 2.3.1. One-dimensional flow solver

The solutions of pressure and flow waveforms presented here were obtained using the 1D flow solver STARFiSh [19]. The hyperbolic partial differential equations for blood flow in compliant vessels are written in terms of pressure and flow variables (*P*, *Q*):

2.1a $$\frac{\partial A}{\partial P}\frac{\partial P}{\partial t}+\frac{\partial Q}{\partial x}=0$$

and

2.1b $$\frac{\partial Q}{\partial t}+\frac{\partial Q^{2}/A}{\partial x}=-\frac{A}{\rho }\frac{\partial P}{\partial x}+\frac{\;f}{\rho },$$

and solved using the explicit MacCormack scheme [20]. Here, *t* is the time, *x* is the axial coordinate, *f* is the frictional term and is given by −2(*ζ* + 2)*μπU*, where *ρ* is the density (1060 kg m^−3^), *μ* is the viscosity of blood (3.5 mPa s), *A* is the cross-sectional area and *U* is the cross-sectional averaged velocity. The following velocity profile was prescribed:

2.2 $$u(x,\xi ,t)=U(x,t)\frac{\zeta +2}{\zeta }\left[1-{\left(\frac{\xi }{r}\right)}^{\zeta }\right],$$

where *r*(*x*, *t*) is the lumen radius, *ξ* is the radial coordinate and *ζ* = 9 is the polynomial order. At arterial connections compatibility of propagating characteristic variables were enforced [7] in addition to conservation of mass and a coupling equation for the pressure, i.e.:

2.3*a* $$\sum_{i=1}^{N}Q_{i}=0$$

and

2.3*b* $$P_{1}+\frac{\rho }{2}U_{1}^{2}=P_{i}+\frac{\rho }{2}U_{i}^{2}+\Delta P\;i=2,\ldots ,N,$$

where *N* is the number of vessels in the connection, and Δ*P* is an additional pressure loss which was set equal to zero for normal connections. At arterial stenoses, the flow regime is 3D and the 1D assumptions no longer hold. Stenotic regions were thus removed and treated as junctions with *N* = 2, however, now with an additional experimental-based pressure loss term given by Liang *et al*. [21]:

2.4 $$\Delta P=K_{\mathrm{visc}}Q+K_{\exp}Q|Q|,$$

where the viscous, *K*_visc_ and expansion, *K*_exp_ coefficients were calculated based on geometrical features, as described in [21].

The pressure–area relation assumes thin-walled elastic vessels and can be derived from Laplace’s Law:

2.5 $$P=P_{\mathrm{dia}}+\frac{\beta }{A_{d}}(\sqrt{A}-\sqrt{A_{d}}),\;\beta (x)=\frac{4}{3}\sqrt{\pi }Eh,$$

where *P*_dia_ is the diastolic pressure with corresponding cross-sectional area *A*_d_, *E* is the elastic modulus and *h* is the thickness of the vessel wall. The stiffness parameters *E h* are related to the pulse wave velocity *c* and have been obtained using the relation [22]:

2.6 $$c_{d}^{2}=\frac{2}{3\rho }\frac{Eh}{r_{d}}=\frac{2}{3\rho }[k_{1}\exp(k_{2}r_{d})+k_{3}],$$

where *r*_d_ is the radius at diastolic pressure, and the values for *k*_1_, *k*_2_ and *k*_3_ were set to 3 × 10^6^ g s^−2^ cm^−1^, −9 cm^−1^ and 33.7 × 10^4^ g s^−2^ cm^−1^ for systemic arteries and 20 × 10^6^ g s^−2^ cm^−1^, −22.5 cm^−1^ and 86.5 × 10^4^ g s^−2^ cm^−1^ for coronary arteries, respectively [9].

#### 2.3.2. Boundary conditions

For the 96-artery model, inflow boundary conditions (prescribed flow rate *Q*) and outflow boundary conditions (three-element Windkessel models, WK3) and all other parameters were adapted from Mynard & Smolich [9]. For the coronary network, the proximal pressure tracing was prescribed at the aortic root. In contrast to systemic arteries, coronary arteries experience increased impedance during systole due to the contraction and increased pressure in the left ventricle. To account for this effect, a lumped parameter WK model WK_cor_ was used at coronary outlets [23]. A schematic of the model is shown in figure 9 in appendix A.1 and the *a priori* computed left ventricle pressure waveform is shown in figure 8. The left ventricle pressure waveform was obtained by coupling a varying elastance (VE) heart model to a WK3 model [24], and further by parameter optimization to minimize the discrepancy between *P*_p_ and *P*_ao_, where *P*_ao_ is the aortic pressure resulting from the VE-WK3 model. The total arterial resistance, *R*_tot_ was estimated from CO, mean arterial pressure, $\bar{P_{p}}$ and outflow WK pressure, *P*_out,WK_ (5 mmHg) according to Ohm’s Law. Total arterial compliance was estimated from the VE-WK3 model. About 4.5% of CO was assumed to supply coronary arteries and used to estimate total coronary resistance and compliance, and was further distributed among coronary outlets according to Murray’s Law [25]. Simulation of a hyperaemic state is necessary for FFR calculations. Hyperaemia was modelled by reducing the resting resistance of the coronary outlets by a factor *α*. The value of *α* was based on the work of Uren *et al.* [26] who studied myocardial blood flow and resistance in relation to the severity of coronary stenosis, and was set to 3 for ‘healthy’ outlets, and to 1.25 for outlets distal of the coronary stenosis. For details see appendix A.2.1.

### 2.4. Network reduction

**Figure 2.**
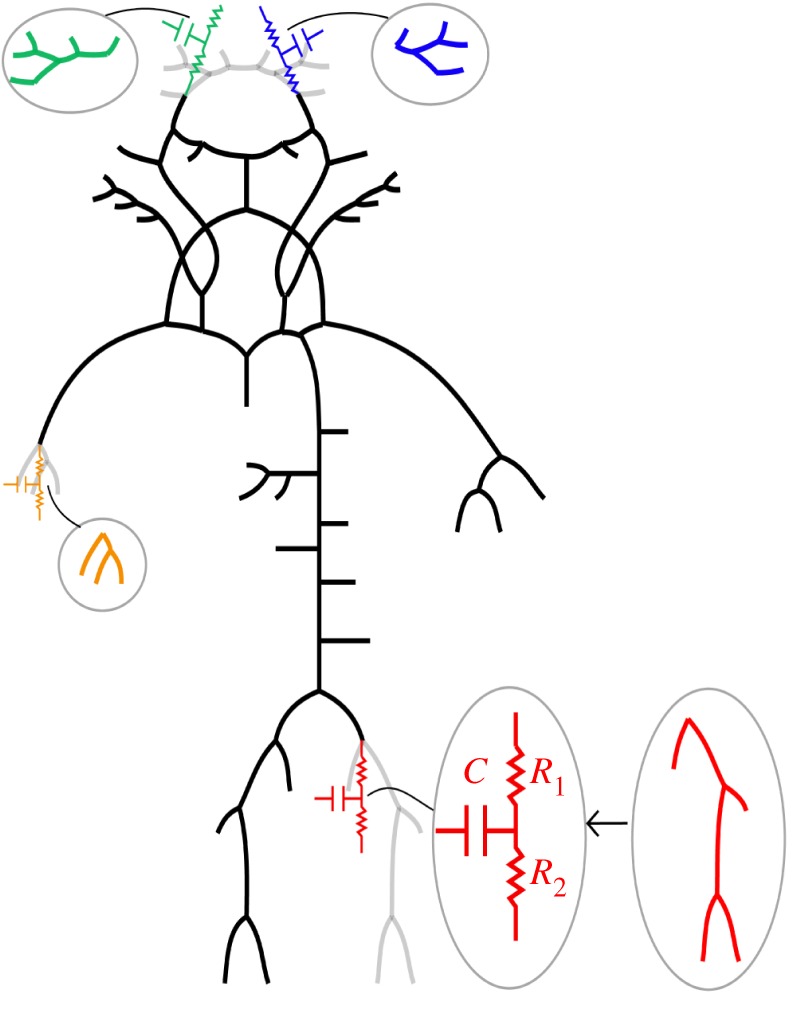
An example of a reduced network that has been obtained from the baseline model in figure 1 by lumping 1D model segments into WK3 models. Lumped 1D model segments are shown in colour.

Network reduction involves lumping distributed 1D segments into 0D parameter models, specifically WK models, intended to represent the same physical problem. Each WK model represents all arteries situated distal of the point of interest with resistance elements and capacitors in series and parallel, as visualized in figure 2.

#### 2.4.1. Method 1, algebraic estimation of lumped parameters

Here, we present a method for network reduction which was adapted from Epstein *et al*. [13]. The method was described and applied on a baseline network only including bifurcations. In this work, we have used a different way of estimating the lumped resistance and compliance that can also be applied on networks containing loops and anastomosis. We have also expanded the procedure to account for arterial stenoses.

##### 2.4.1.1. Estimation of lumped resistance

The linearized version of equations (2.1*a*) and (2.1*b*) can be written in terms of the steady-state variables $\bar{P}$, $\bar{Q}$ and $\bar{A}$:

2.7*a* $${\bar{Q}}_{\mathrm{in}}={\bar{Q}}_{\mathrm{out}}$$

and

2.7*b* $${\bar{P}}_{\mathrm{in}}={\bar{P}}_{\mathrm{out}}+\int_{0}^{l}\frac{2(\zeta +2)\pi \mu }{{\bar{A}}^{2}}\;\text{d}x,$$

where *l* is the length of the segment, and the subscripts ‘in’ and ‘out’ denote variables at the inlet and outlet of the segment, respectively. Equations (2.7*a*) and (2.7*b*) may then be combined with equations (2.3*a*)–(2.3*b*) and equation (2.5) to form a system of nonlinear algebraic equations. The system was solved iteratively by employing Picard linearization. $\bar{P}$ and $\bar{Q}$ is in such an estimate of the time average of *P*(*t*) and *Q*(*t*), and once solved for, resistance may be estimated anywhere in the network using Ohm’s Law:

2.8 $$R=\frac{\bar{P}-P_{\mathrm{out},\mathrm{WK}}}{\bar{Q}}.$$

##### 2.4.1.2. Estimation of lumped compliance

We can estimate the compliance (*C*_v_) of a vessel by integrating over the length of the 1D model segment [13]:

2.9 $$C_{v}=\frac{K_{1}}{\rho },\;K_{1}=\int_{0}^{l}\frac{\bar{A}}{{\bar{c}}^{2}}\;\text{d}x.$$

**Figure 3.**
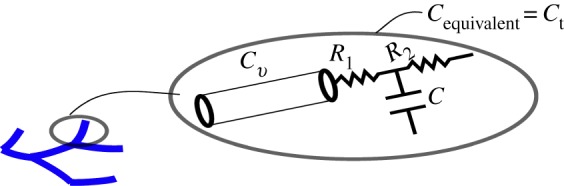
Illustration of an arterial 1D model segment coupled to a WK3 model that may be lumped into an equivalent compliance *C*_t_ according to equation (2.10). (Online version in colour.)

Furthermore, we estimated the compliance *C*_t_ of a terminal vessel (figure 3) coupled with a WK3 with proximal resistance, *R*_1_, compliance, *C* and peripheral resistance, *R*_2_ [13]:

2.10 $$C_{t}=\frac{C_{v}R_{2}+C_{v}R_{1}+CR_{2}+C_{v}R_{v}}{R_{2}+R_{1}+R_{v}}.$$

Lumped compliance of terminal vessels coupled to WK_cor_ models (see figure 9 in appendix A.2.1.) with compliances *C*_a_ and *C*_m_ were estimated according to:

2.11 $$C_{t}=C_{v}+C_{a}+C_{m}.$$

The total compliance contribution of vessels distal of a point of truncation was then obtained using equation (2.9) for non-terminal vessels and equation (2.10) or equation (2.11) as appropriate for terminal vessels, together with summation rules for compliances/capacitors in series and parallel. See appendix A.2.1. for details.

##### 2.4.1.3. Lumping vessels distal of a site of truncation

With the lumped resistance, (equation (2.8)) and compliance (equations (2.9)–(2.11)), as defined above we may replace all vessels distal of a point of interest with a WK model. Systemic arteries were replaced by WK3 models in which *R*_1_ was set equal to the characteristic impedance, *Z*_*c*_:

2.12 $$Z_{c}=\frac{\rho \bar{c}}{\bar{A}}.$$

Lumped coronary arteries were replaced by WK_cor_ models and the lumped resistance and compliance were divided among the resistance and compliance parameters of the WK_cor_ model as described in appendix A.2.1.

#### 2.4.2. Method 2, optimization of lumped parameters

Method 1 is based solely on the topology and properties of the baseline model. This means that we can use the method without solving the baseline model. However, the parameters in the WK models that replace the removed vessels are not necessarily the ones that correspond with the least discrepancy between the baseline and reduced networks. This motivates another method which is based on parameter optimization. Since the WK models are lumped models with governing ordinary differential equations (ODEs), we suggest a procedure that treats every truncated site independently. The optimization is thus performed by taking the flow from the 1D solution of the baseline model as given inflow to the WK models, then solving for the unknown pressure. Furthermore, we seek to minimize the error between the pressure obtained by solving the ODE with the corresponding 1D baseline solution. In the following, we explain the procedure for the WK3 model, though it can be easily expanded to other lumped parameter outflow models. Either one, two or all three of *R*_1_, *C* and *R*_2_ were allowed to vary to minimize the error. If only one of *R*_1_ and *R*_2_ was optimized, the total resistance *R*_1_ + *R*_2_ was found from (*P*_avg_ − *P*_out,WK_)/*Q*_avg_, where *P*_avg_ and *Q*_avg_ are the time-averaged pressure and flow from the 1D baseline solutions. The method may be summarized in the following steps:

(1) Calculate the flow and pressure waveforms of the 1D baseline model.
(2) Locate the sites where WK3 models will replace distal vessels.
(3) Calculate values of *R*_1_ + *R*_2_ from *P*_avg_, *Q*_avg_, and *C* using Method 1 (§2.4.1).
(4) Use the flow from the 1D baseline model as given inflow of the WK3 ODE, with parameters *R*_1_, *C* and *R*_2_.
(5) Choose parameters to be optimized and use parameters from point 3 otherwise and as initial guess.
(6) Solve the WK3 ODE for the unknown pressure, *P*_WK3_.
(7) Find the parameters that minimize the discrepancy between *P*_WK3_ and the corresponding pressure waveform from the solution of the 1D baseline model. We used the average relative error, calculated by equation (2.13*a*) as the measure of discrepancy.

Based on a parameter correlation and identifiability analysis, we chose to optimize on the subset of parameters ([*θ*_1_, *θ*_2_] = [*R*_1_/*R*_2_, *C*]), where *R*_1_ + *R*_2_ was kept constant. See appendix A.3.2. for details.

### 2.5. Error metrics

The following error metrics were used to compare pressure and flow waveforms obtained from the baseline (B) and reduced (R) models:

2.13*a* $$\epsilon_{P,\mathrm{avg}}=\frac{1}{N_{t}}\sum_{i=1}^{N_{t}}\left|\frac{P_{i}^{R}-P_{i}^{B}}{P_{i}^{B}}\right|,\;\epsilon_{Q,\mathrm{avg}}=\frac{1}{N_{t}}\sum_{i=1}^{N_{t}}\left|\frac{Q_{i}^{R}-Q_{i}^{B}}{{\text{max}}_{j}(Q_{j}^{B})}\right|,$$

2.13*b* $$\epsilon_{P,\mathrm{sys}}=\frac{\left|P_{\mathrm{sys}}^{B}-P_{\mathrm{sys}}^{R}\right|}{P_{\mathrm{sys}}^{B}},\;\epsilon_{P,\mathrm{dia}}=\frac{\left|P_{\mathrm{dia}}^{B}-P_{\mathrm{dia}}^{R}\right|}{P_{\mathrm{dia}}^{B}},$$

2.13*c* $$\epsilon_{PP}=\frac{\left|PP^{B}-PP^{R}\right|}{PP^{B}},$$

2.13*d* $$\epsilon_{P,\mathrm{aug}}=\frac{\left|\{P_{\mathrm{sys}}^{B}-P_{\mathrm{infl}}^{B}\}-\{P_{\mathrm{sys}}^{R}-P_{\mathrm{infl}}^{R}\}\right|}{PP^{B}}$$

and

2.13*e* $$\epsilon_{\mathrm{iFR}}=|{\text{iFR}}_{B}-{\text{iFR}}_{R}|,$$

where *N*_*t*_ is the number of time points in a cardiac cycle, *i* represents a certain time point with corresponding baseline, $P_{i}^{B}$ and reduced, $P_{i}^{R}$ pressure and flow ($Q_{i}^{B}$, $Q_{i}^{R}$), respectively. *ε*_*Q*,avg_ was normalized by the maximum flow of the baseline model over one cardiac cycle, ${\text{max}}_{j}\;(Q_{j}^{B})$, to avoid division by numbers close to zero. The maximum (*P*_sys_) and minimum pressure (*P*_dia_) was used to calculate the systolic (*ε*_*P*,sys_), and diastolic (*ε*_*P*,dia_) error, respectively. The pulse pressure, *PP* is defined as *P*_sys_ − *P*_dia_. *ε*_*PP*_ is the error in pulse pressure and *ε*_*P*,aug_ is the error in augmentation pressure, both normalized by the pulse pressure. $P_{\mathrm{infl}}^{B}$ is the pressure at the inflection point in early systole [27]. *ε*_iFR_ is the difference between predicted iFR from baseline and reduced model.

### 2.6. Application to different physiological and pathological states

The parameters for the baseline 96-artery model were based on data from healthy, young adults [9]. In this part of the study, however, we re-parametrized a series of optimal networks to represent (1) normal ageing, (2) a pathological state of aortic coarctation and (3) states of different heart rate, ejection time and stroke volume. We note that no information from the baseline model was used to re-parametrize the reduced models.

#### 2.6.1. Normal ageing

Normal ageing was simulated by increasing total arterial resistance by a factor of 1.1, and decreasing total arterial compliance by a factor of 2. Arterial stiffening is most marked in the proximal aorta and its major branches—brachiocephalic, carotid, subclavian [28]. The stiffness parameter *β* for these arterial segments was increased by a factor of 2.5, whereas it was increased by a factor of 1.5 for all other segments. Finally, the compliance of the WK3 models were modified so that the total arterial compliance (sum of WK3 compliance of terminal segments and integrated 1D compliance) was decreased by a factor of 2. The total arterial resistance was modified by increasing the peripheral resistance in all outflow WK3 models. See appendix A.5 for details.

#### 2.6.2. Aortic coarctation

Aortic coarctation was simulated by introducing a 1 cm long, 50% diameter stenosis in the thoracic aorta. This corresponds to segment Id 18 in the electronic supplementary material.

#### 2.6.3. Heart rate, ejection time and stroke volume

Heart rate, ejection time and stroke volume were modified according to the study by Weissler *et al.* [29]. They studied relationships between left ventricular ejection time, ET, stroke volume, SV and heart rate, HR, in normal individuals. We modified the original aortic inflow curve for the 96-artery model to represent the two extreme cases in terms of HR in their study (HR: 56 bpm, ET: 0.315 s, SV: 106 ml and HR: 120 bpm, ET: 0.2 s, SV: 44 ml). For the latter, total arterial resistance was increased by a factor of 1.67 and compliance halved (effecting the distributed parameters as described for normal ageing), in order to obtain physiological pressure waveforms.

## 3. Results

### 3.1. Comparison of Method 1 and Method 2 for network reduction

**Figure 4.**
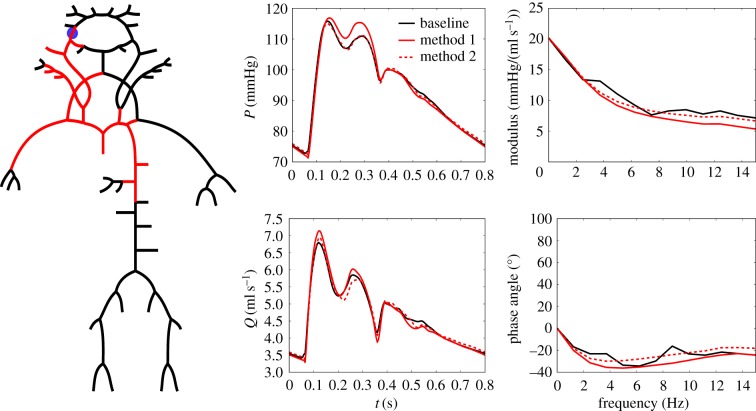
Comparison of Method 1 and Method 2 for network reduction. Baseline model in black and reduced models in red together with pressure and flow waveforms at the inlet of the right internal carotid artery. Impedance modulus and phase angle are also shown.

Figure 4 shows the 96-artery model (black) reduced to a 25-artery model (red). Solution of pressure and flow waveforms at the inlet of the right internal carotid artery, obtained from the baseline model and both methods for network reduction, are also shown. Method 1 overestimated internal carotid pressure in mid systole (*ε*_*PP*_ was 6.2% for Method 1 and 0.2% for Method 2). Furthermore, Method 2 captured the overall shape of pressure and flow waveforms better than Method 1. Average errors, *ε*_*P*,avg_ between full and reduced models were 1.45% for Method 1 and 0.57% for Method 2. Similarly, *ε*_*Q*,avg_ was 1.47% and 1.16%, respectively. Figure 4 also shows the impedance modulus and angle for the site of interest, calculated in the frequency domain as explained in [30].

### 3.2. Framework for optimizing topological complexity

**Figure 5.**
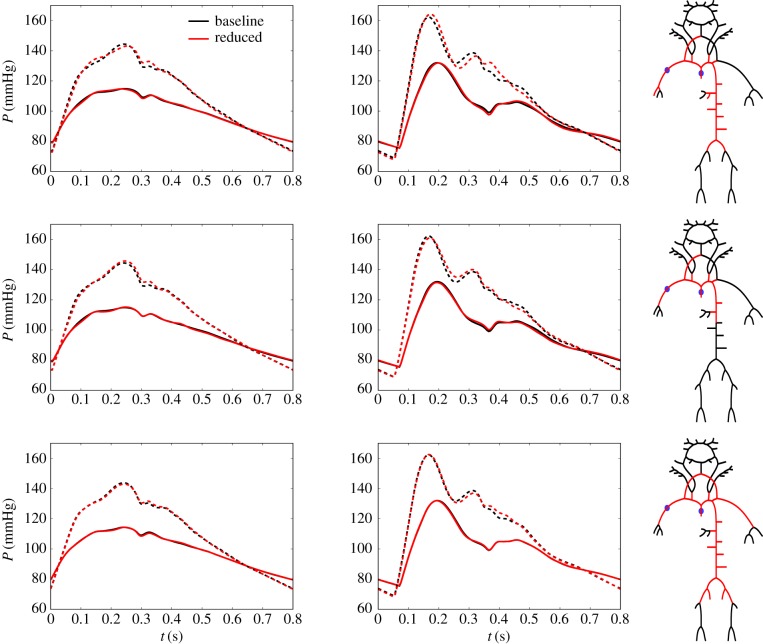
Optimal networks and corresponding waveforms (solid lines) obtained from the 96-artery baseline model with pressure at midpoint of ascending aorta (left) and right brachial artery (middle) set as quantities of interest. In the top and middle rows, an averaged (of the two quantities of interest) error threshold of *ε*_*P*,sys_ + *ε*_*P,*dia_ less than 0.3% (top row) and 1.0% (middle row) was used. In the last case, an error threshold of *ε*_*P*,aug_ + *ε*_*PP*_ less than 0.7% was used for the aorta. Dashed waveforms correspond to simulations of normal ageing as described in §2.6.

**Figure 6.**
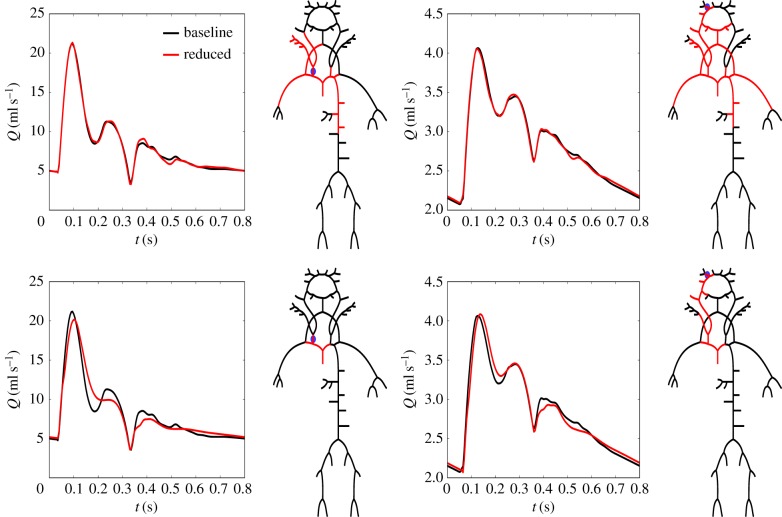
Optimal networks obtained from the 96-artery baseline model with the flow at the distal end of the right carotid artery set as quantity of interest (left panel) and with 0.9 and 3.4% average error thresholds, respectively. The right panel show results when the flow at the proximal end of the right middle cerebral artery was set as quantity of interest and with 0.6 and 1.6% average error thresholds, respectively.

A summary of the quantities of interest, error metrics and values for the network reduction framework applied on the 96-artery model is given in table 1. Here, error metrics are also presented for the cases where parameters were altered to simulate different physiological and pathological states (see §2.6). References to associated figures are also given. In particular, a threshold based on *ε*_*P*,sys_ + *ε*_*P*,dia_ at the aorta and brachial artery was used in the top two examples in figure 5. The waveforms for the baseline model and optimal reduced networks are shown in solid lines, and the dashed lines represent the case when the models were altered to represent normal ageing. In the last example, a threshold based on augmentation and pulse pressure was used ($\epsilon_{P,\mathrm{aug}}+\epsilon_{PP}<0.7\text{\%}$). Furthermore, in order to ensure that interaction between different regions in the network and that pressure propagation are correctly captured throughout the larger systemic arteries, a threshold based on pressure waveforms at four locations was used in figure 13 in appendix A.5. Here, the average *ε*_*P*,avg_ for the aortic root, common carotid, brachial and femoral artery pressure waveforms was required to be less than 0.4%. Additionally, results are shown for *ε*_*Q*,avg_ less than 0.9 and 3.4% for the right common carotid artery and *ε*_*Q*,avg_ less than 0.6 and 1.6% for the middle cerebral artery in figure 6. Method 2 (§2.4.2) was used to reduce the networks in all these cases.

**Table 1.** Summary of results from applying the framework outlined in §2.1, on the 96-artery baseline model. For cases where there are more than one quantity of interest, the final error was calculated as the average of the error for the individual quantities. Ref. denotes the reference case, and the threshold used for the optimization is given in brackets. The errors are also shown for states of normal ageing, aortic coarctation (coarc.) and for the two aortic inflow curves as defined in §2.6. All errors are in percentage. The associated figure numbers are referenced below the error, where available.

quantity of interest	no. of arteries	error- metric	ref.	ageing	coarc.	inflow 1	inflow 2
aortic and brachial pressure	29	*ε*_*P*,sys_ + *ε*_*P*,dia_	0.25 (0.3) 5	2.26 5	0.37 —	0.14 —	1.58 —
aortic and brachial pressure	15	*ε*_*P*,sys_ + *ε*_*P*,dia_	0.92 (1.0) 5	1.0 5	0.27 —	1.13 —	1.3 —
aortic pressure	31	*ε*_*PP*_ + *ε*_*P*,aug_	0.68 (0.7) 5	0.97 5	1.91 —	0.41 —	1.27 —
aortic and brachial and carotid and femoral pressure	31	*ε*_*P*,avg_	0.33 (0.4) 13	0.47 13	0.24 15	0.41 16	0.85 16
carotid flow	25	*ε*_*Q*,avg_	0.87 (0.9) 6	1.53 14	1.09 —	0.81 17	2.97 17
carotid flow	5	*ε*_*Q*,avg_	3.35 (3.4) 6	5.2 14	— —	2.93 17	6.3 17
r. middle cerebral flow	38	*ε*_*Q*,avg_	0.59 (0.6) 6	1.13 14	0.66 —	0.66 17	2.88 17
r. middle cerebral flow	15	*ε*_*Q*,avg_	1.58 (1.6) 6	2.64 14	— —	1.72 17	4.38 17

**Figure 7.**
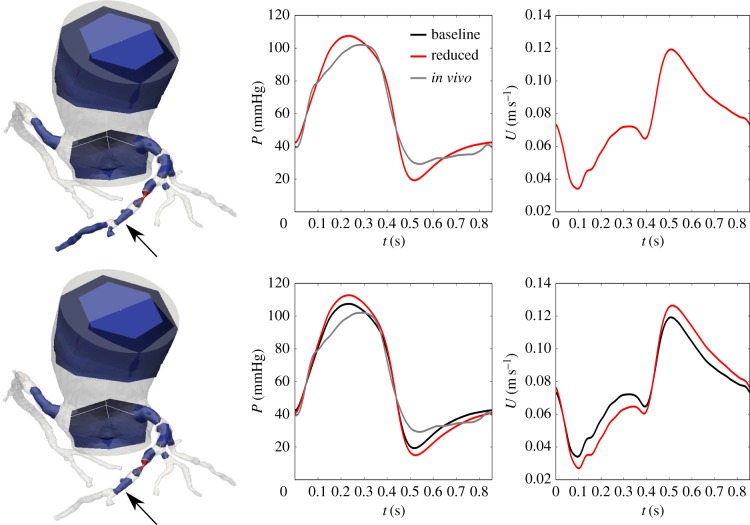
Optimal networks obtained from the coronary network with difference in iFR set as quantity of interest, with *ε*_iFR_ < 0.00012 (top) and *ε*_iFR_ < 0.04 (bottom). Results are visualized through the distal pressure waveform. *In vivo* pressure waveform and predicted velocity waveforms are also shown. The arrow indicates the location of the waveforms, and the red section a significant stenosis.

**Figure 8.**
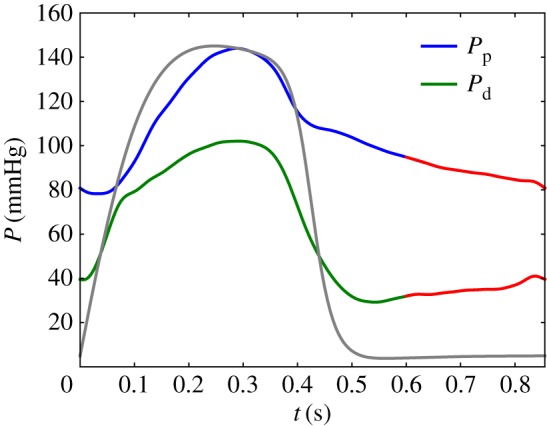
Proximal *P*_p_ and distal *P*_d_ pressure waveforms obtained during invasive angiography, together with a computed left ventricle pressure waveform in grey. The last 30% of the cardiac cycle is highlighted in red and was used to compute the instantaneous wave-free ratio, iFR, a drug-free index of the significance of the stenosis.

**Figure 9.**
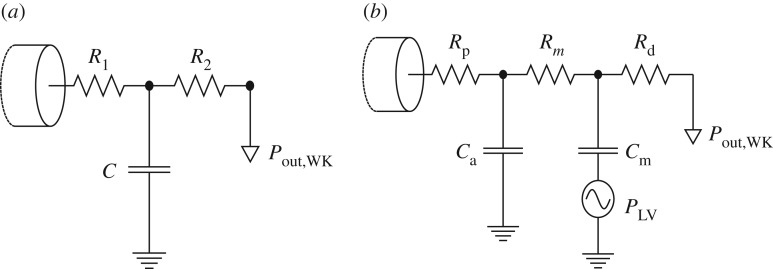
Schematic of the two lumped parameter models used in this work, WK3 model (*a*) and WK_cor_ (*b*). *R*_1_, *R*_2_, *R*_p_, *R*_*m*_ and *R*_d_ are resistance parameters, *C*, *C*_a_ and *C*_m_ are compliance parameters and *P*_LV_ and *P*_out,WK_ are left ventricle and outflow windkessel pressures, respectively.

In the top part of figure 7, *ε*_iFR_ was set to 0.033, which is the standard deviation of repeated iFR measurements, according to the study by Johnson *et al.* [15]. The results are visualized through the distal pressure waveform, *P*_d_. All side branches except those distal of the measured location can be replaced by lumped WK_cor_ models with no visible effect and with *ε*_iFR_ < 0.000012. If the threshold is increased to 0.04 the network can be reduced to its most simplistic realization, as visualized in the bottom part of the figure. The predicted velocity and the *in vivo* pressure waveforms are also shown. iFR was measured to 0.40, whereas the predicted value was 0.42 for the baseline network, and 0.42 and 0.38 for the reduced networks, respectively. For FFR, the measured value was 0.52, whereas the predicted value was 0.48 for the baseline network and both of the reduced networks. Method 1 (§2.4.1) was used to reduce the coronary networks.

## 4. Discussion

In this study, we have presented a novel approach which optimizes the number of arterial segments for 1D blood flow models. We have illustrated the framework on a 96-artery and a coronary baseline model, and two methods for network reduction have been incorporated: a purely algebraic method (Method 1, §2.4.1) and a novel method based on optimization (Method 2, §2.4.2).

### 4.1. Comparison of methods for network reduction

A major difference in the waveforms obtained from Method 1 and Method 2 may be seen in the systolic part of the cycle, where the pressure obtained using Method 1 was overpredicted. This was observed as a general distinction between the two methods, and is exemplified in figure 4. However, the diastolic phase is very similar, indicating that the discrepancy is not a result of differences in the values of compliance in the WK3 models. The diastolic decay of pressure can be approximated by an exponential function, with an exponent given by the product of the peripheral resistance (*R*_2_) and the compliance (*C*) [31]. Thus changes in the compliance directly effect the diastolic shape, whereas changes in *R*_1_ only have secondary effect. On the other hand, *R*_1_ has a direct effect on the systolic part of the cycle. Inspection of the values used for the proximal resistance in the WK3 models revealed that *Z*_*c*_ (Method 1) was in general higher than *R*_1,opt_ (Method 2) for the larger systemic arteries. The addition of the characteristic impedance to the original two-element WK model was based on frequency analysis of modulus and phase of the input impedance along the aorta. By including the characteristic impedance, the input impedance modulus of the modified WK matched *in vivo* measurements at high frequencies [2,31]. We also observed (not shown here) better matching of the modulus at the aorta for high frequencies, between baseline and reduced models obtained with Method 1 than with Method 2; however, the same is not true for this more distal location (internal carotid). Impedance phase, on the other hand, was captured better by Method 2 for some frequencies (particularly between 5 and 7 Hz), as can also be seen in the phase of the first minima of the flow waveform (≈ 6 Hz). Minimization of high-frequency oscillations has also been an incentive for using matched (*R*_1_ = *Z*_c_) WKs as outflow BC’s in 1D blood flow models [12]. However, the price to pay is an overprediction of pressure in systole.

### 4.2. Optimization of topological complexity

#### 4.2.1. Central and larger systemic artery pressure waveforms

Pressure measured with a cuff and sphygmomanometer in the brachial artery is used routinely and accepted as an important predictor of future cardiovascular risk. However, studies indicate that central blood pressure (CBP) relates more strongly to cardiovascular events [32]. Systolic and pulse pressures are amplified as the pulse wave propagates through the larger systemic arteries. This amplification may vary significantly among subjects [32], making it difficult to map measurements of pressure at more peripheral sites directly to CBP.

Although it is still unclear if routine measurement/estimation of CBP will provide significantly improved risk stratification [33], the 1D nonlinear equations for blood flow can be used to investigate pulse wave amplification [8,34,35]. In previous studies, the topology of the 1D model was chosen *ad hoc*. Our novel framework provides a mathematical approach to determine the optimal topology to study pulse wave amplification from the aortic root to the brachial artery.

The results presented in the first two rows of figure 5 indicate that inclusion of detailed descriptions of upper and lower limbs are not needed in order to study pulse wave amplification from the aortic root to the brachial artery. Moreover, the entire cerebral circulation can be replaced by WK3 models with negligible effects on aortic and brachial pressure waveforms. This is reasonable since these are relatively small and stiff arteries for which the behaviour is well captured by WK3 models [3]; however, it is important to note that the proximal part of the aorta, which accounts for about 50% of total systemic compliance, needs to be kept in the reduced 1D model.

**Figure 13.**
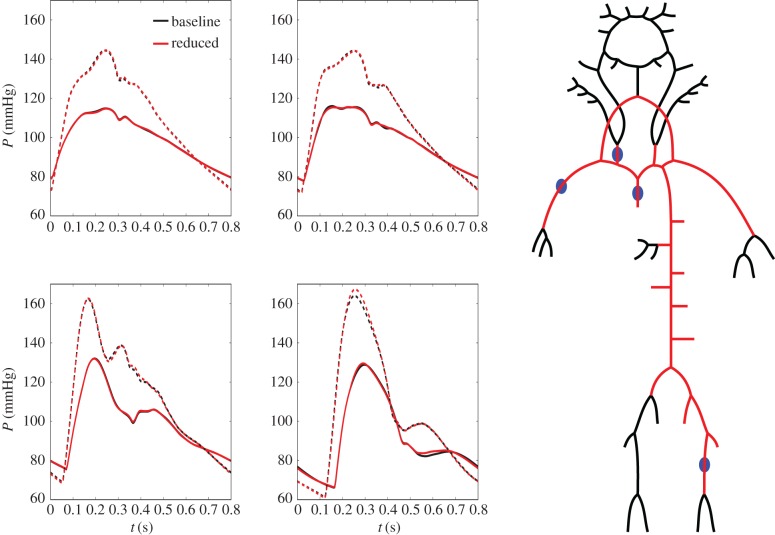
Optimal network obtained from the 96-artery baseline model with pressure at midpoint of ascending aorta (top left), right common carotid artery (top middle), right brachial artery (bottom left) and left femoral artery (middle bottom) set as quantities of interest. The average deviation at these four locations was used as threshold, and set to 0.4% average error. Dashed waveforms represent the case when baseline and reduced networks were re-parametrized to simulate normal ageing (see §2.6).

Both pulse pressure and augmentation pressure, and their relation (augmentation index) is associated with cardiovascular risk [36]. Even though the aortic pressure waveforms obtained by the reduced models in the top two examples in figure 5 captured the pulse pressure very well, some subtle deviations are visible in the systolic part of the waveforms. This could have an effect on the calculated augmentation pressure, and thus also on evaluations of cardiovascular risk. In the last example in figure 5, an error threshold of *ε*_*PP*_ + *ε*_*P*,aug_ of 0.7% at the aorta, was used, and results indicate that this 31-artery model captures the most important features of wave propagation for central aortic pressure. A similar model was found when a combined threshold of average *ε*_*P*,avg_ of 0.4% was set for four arterial sites; midpoint of ascending aorta, right common carotid artery, right brachial artery and left femoral artery, as illustrated in figure 13 in appendix A.5. This network was also able to capture waveform features with good qualitative and quantitative precision when the model was re-parametrized to model different physiological and pathological states.

#### 4.2.2. Carotid and cerebral circulation

**Figure 14.**
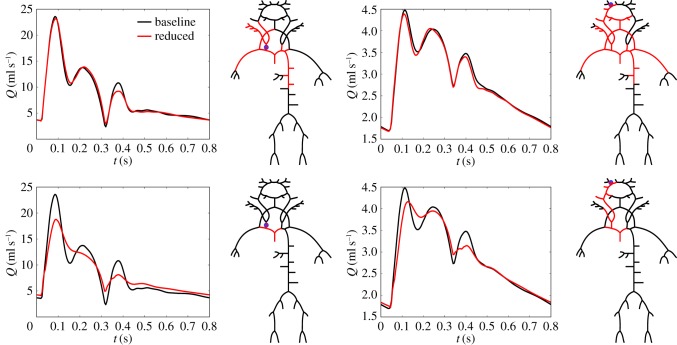
Optimal reduced networks for flow at the distal end of the right carotid artery (left panel) and proximal end of the right middle cerebral artery (right panel). The networks are the same as shown in figure 6; however, parameters were altered in order to represent normal ageing as described in §2.6.

In the study by Reymond *et al.* they compared carotid flow predictions with and without description of the cerebral circulation and stated that a detailed description was necessary in order to produce physiological correct waveforms. Our results, on the other hand, indicate that the entire cerebral circulation can be appropriately lumped into WK3 models effecting only the diastolic part of the flow waveforms and with $\epsilon_{Q,\mathrm{avg}}<0.9\text{\%}$, as shown in figure 6. Furthermore, by increasing the threshold to 3.4% the network is reduced to a very simplistic model including only five arterial segments. Though the overall features are represented in this five-artery model, the arterial tree is truncated close to the carotid artery and will thus be more influenced by the WK3 models. High-frequency details are not described well by the three-element WK [31], which in this case is visible through the smoothing of the second and third peaks of the flow waveform. Such errors were magnified when the model was transformed to represent normal ageing, as visualized in figure 14.

Figure 6 also shows results with flow rate at the inlet of the right middle cerebral artery set as the quantity of interest. This site is located more distal than the other quantities of interest studied in this work, and as can be seen in the case where a threshold of $\epsilon_{Q,\mathrm{avg}}<0.6\text{\%}$ was considered, the circle of Willis can be ‘broken’ and represented by WK3 models without altering the flow waveform significantly. Furthermore, the arterial tree can be truncated in close proximity to the middle cerebral artery without introducing significant constraints on the solution, more so than was the case for the right common carotid artery. This is attributed to the fact that the flow in this region is more dominated by frictional forces resulting in pressure and flow waveforms that are of similar shape and phase and can be more readily described by the WK3 model. Moreover, by increasing the threshold to $\epsilon_{Q,\mathrm{avg}}<1.6\text{\%}$ more of the larger systemic arteries may also be lumped, resulting in very simplistic descriptions of the arterial network that were still able to capture the main features of the flow waveform in the middle cerebral artery. For this model, however, errors were magnified when parameters were altered to represent different physiological states, indicating that having a reasonably complete description of the larger arteries is more important than including the nearby system of 1D model arteries.

Blood flow can be measured non-invasively by ultrasound in both the carotid and middle cerebral arteries; however, there are many sources of uncertainty and standard errors of measurements are normally higher than 10% [37]. In comparison, the modelling errors introduced by applying network reduction to obtain simpler descriptions of the arterial system were smaller.

#### 4.2.3. Coronary pressure waveforms

Figure 7 shows the results from applying our methodology on the patient-specific coronary network. The model can be reduced to its most simplistic realization while still keeping the error for the predicted iFR on a level which is comparable with the standard deviation of repeated iFR measurements. The differences in predictions of FFR between baseline and reduced models were even smaller, and in fact smaller than the significant figures used in clinical decision-making. This is attributed to the fact that, unlike iFR, FFR is a cardiac cycle averaged quantity. Our approach for network reduction maintained the correct resistance throughout the domain, and thus also average flow and pressure distributions. The limited resolution of CCTA imaging contributes a layer of uncertainty since only features larger than approximately 1.0 mm can be resolved [38]. However, our results indicate that one should not necessarily strive to segment arteries down to this limit.

## 5. Concluding remarks

Our results have shown that to capture important features of the aortic pressure waveform, such as timing and shape of reflected waves, pressure augmentation and pulse pressure, a model with all aortic segments, but close to minimal description of the head and lower and upper limb arteries is sufficient. Furthermore, a detailed description of the cerebral circulation is not needed in order to capture physiologically correct waveforms in the common carotid and middle cerebral arteries. Even though our framework for network reduction was performed on a single set of parameters representing a normal physiological state, waveform features were also captured with good qualitative and quantitative precision when the models were re-parametrized to simulate different physiological and pathological states.

Our approach is targeted at computational models of the cardiovascular system, however, it should also be useful for the design of *in vitro* haemodynamic experiments. Such physical models are attractive tools for fundamental research on pulse wave propagation [30,39], and also play a key role in validating computational models [6]. Through further work, one could also imagine the relevance of our approach in the design of multi-scale models of the cardiovascular system, e.g. hybrid 3D–1D–0D models.

## Supplementary Material

Supplementary material for optimization of complexity for arterial blood flow models

## Figures and Tables

**Figure 10.**
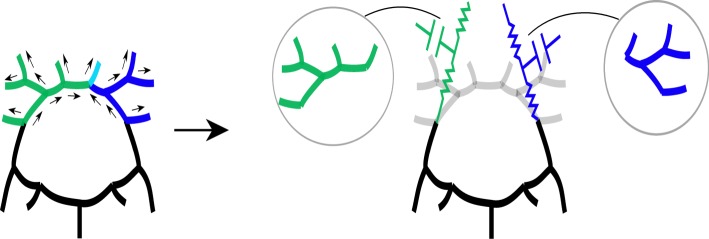
Arrows indicate the direction (not magnitude) of $\bar{Q}$, and also which arteries are lumped into WK3 models for two selected sites of truncation: the left and right internal carotid arteries.

**Figure 11.**
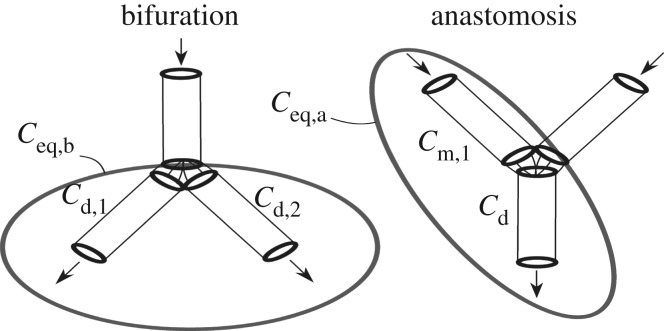
Compliance contribution from vessels in bifurcations and anastomosis used in equation (A6*a*) and equation (A6*b*). Arrows indicate the direction of flow.

**Figure 12.**
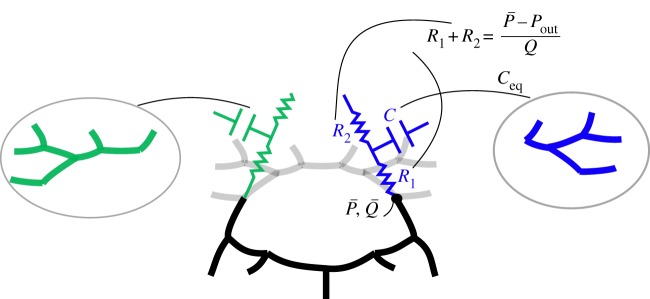
Illustration of vessels distal of points of truncation lumped into WK3 models.

**Figure 15.**
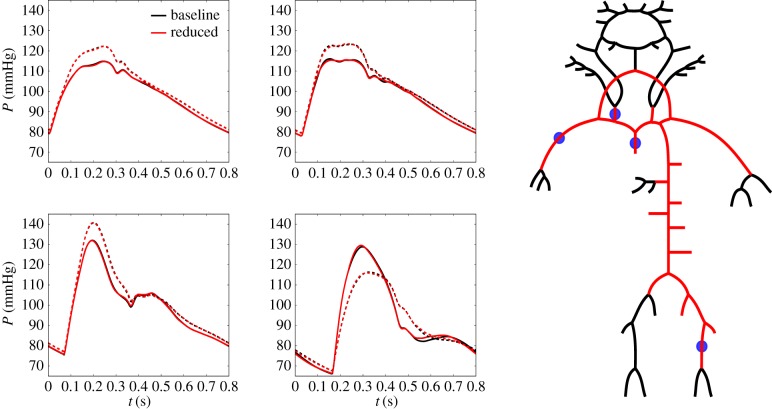
Optimal reduced network for ascending aorta (top left), right common carotid artery (top middle), right brachial artery (bottom left) and left femoral artery (middle bottom). The network is the same as shown in figure 13; however, a pathological state of aortic coarctation (dashed lines) as described in §2.6 is also shown.

**Figure 16.**
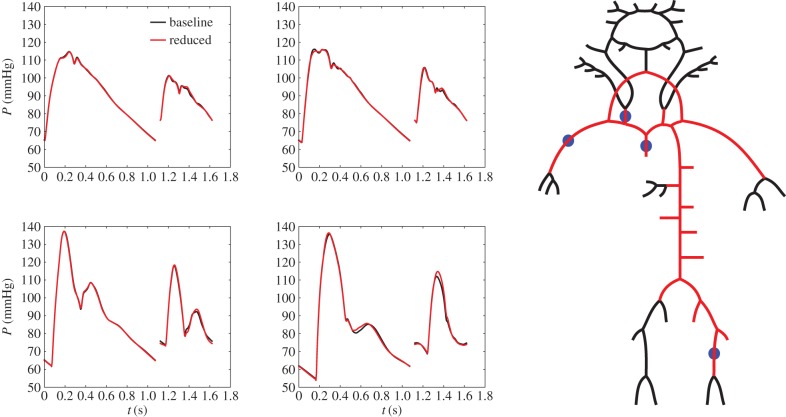
Optimal reduced network for ascending aorta (top left), right common carotid artery (top middle), right brachial artery (bottom left) and left femoral artery (middle bottom). The network is the same as shown in figure 13; however, results are shown for two different inlet waveforms as described in §2.6.

**Figure 17.**
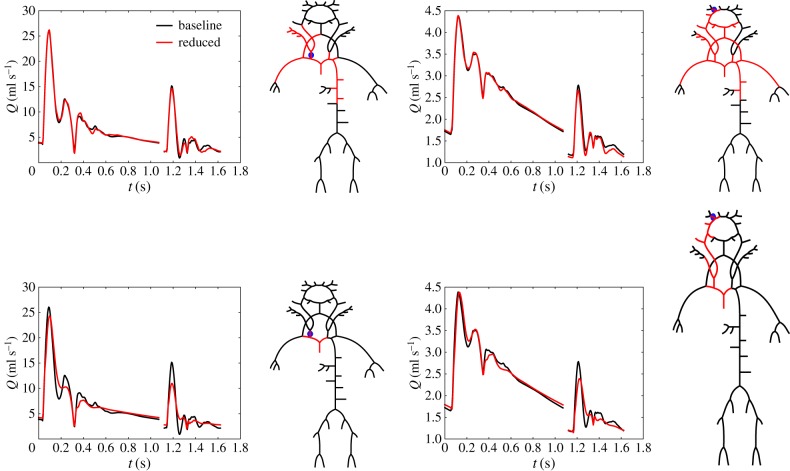
Optimal reduced networks for flow at the distal end of the right carotid artery (left panel) and proximal end of the right middle cerebral artery (right panel). The networks are the same as shown in figure 6; however, results are shown for two different inlet waveforms as described in §2.6.

## Data accessibility

The analyses performed in this study were based on the open-source 1D flow solver, STARFiSh (https://www.ntnu.no/starfish). Code and data to reproduce the analyses will be available at https://github.com/Biomechanics-NTNU, shortly. Until then, the files are hosted at the temporary repo https://github.com/Fredf10/starfish_networkReduction_tmp. Raw data in the format produced by STARFiSh, with a size of approximately 130 GB are available upon request.

## Appendix A. Material and methods

**A.1. *In vivo* data: measurement and post-processing**

Proximal *P*_p_ and distal (of a coronary stenosis) *P*_d_ pressure tracings were available from a patient with positive findings of coronary artery disease. Pressure tracings were obtained by insertion of a Volcano pressure wire during invasive angiography. *P*_p_ and *P*_d_ together with a computed (see §A.2.1) left ventricle pressure waveform (grey) are shown in figure 8. CO was measured using transthoracic Doppler echocardiography.

**A.2. Numerical formulation**

**A.2.1. Boundary conditions**

The arterial 1D model segments were terminated with WK3 models (systemic arteries) and WK_cor_ models (coronary arteries). In the latter, the influence from the left ventricle pressure, *P*_LV_ results in a higher coronary impedance in systole. A patient-specific *P*_LV_ was obtained by coupling a varying elastance (VE) heart model with elastance *E*(*t*), volume *V* and intersect volume *V*_0_:

A 1 $$P_{\mathrm{LV}}=E(t)(V-V_{0}),$$

with an aortic pressure *P*_ao_ described by a WK3 model as in [24]. The discrepancy between *P*_ao_ and *P*_p_ was then minimized through parameter estimation. The resulting left ventricle pressure is shown if figure 8. The WK3 and WK_cor_ models, and their coupling with the 1D domain are depicted in figure 9. In the baseline 96-artery model, which only includes systemic arteries, parameters for the outflow WK3 models were adapted from [9]. For the coronary network model, the total arterial resistance and total coronary resistance were estimated by:

A 2 $$R_{\mathrm{tot}}=\frac{{\bar{P}}_{p}-P_{\mathrm{out},\mathrm{WK}}}{\text{CO}}\;\text{and}\;R_{\mathrm{tot},\mathrm{cor}}=\frac{{\bar{P}}_{p}-P_{\mathrm{out},\mathrm{WK}}}{\lambda \times \text{CO}},$$

where λ is the fraction of CO supplying coronary arteries, assumed to be 4.5%. The total arterial compliance, *C*_tot_ was estimated from the VE-WK3 model and total coronary compliance calculated as *C*_tot__,__cor_ = λ*C*_tot_. *R*_tot__,__cor_ and *C*_tot__,__cor_ were further distributed to coronary outlets using Murray’s Law [25]. The total resistance for outlet *j*, *R*_tot__,__cor, j_ was then divided among *R*_p_, *R*_m_ *R*_d_, with fractions 0.01, 0.84, 0.15, respectively, and *C*_tot,cor,*j*_ between *C*_*a*_ and *C*_*m*_ with fractions 0.025 and 0.975, respectively.

The estimated coronary resistance given by equation (A 2) assumes zero resistance in the 1D domain. We therefore used the methods described in §2.4.1.1 to estimate mean flow values, and updated *R*_tot, cor_ until total coronary flow reached the target flow of 4.5% of CO.

**A.3. Network reduction**

**A.3.1. Method 1, algebraic estimation of lumped parameters**

In figure 10, we have separated the circle of Willis from the rest of the 96-artery model to illustrate how network reduction was performed. Here, the network was truncated at two sites. On the left side of the figure, arrows indicate the direction of the calculated mean flow rate $\bar{Q}$ as described in §2.4.1.1, and defines which arterial segments are distal of a site of truncation.

Once this is known the lumped compliance contribution of these vessels may be calculated. We can estimate the compliance (*C*_v_) of a vessel by integrating over the length of the 1D model segment [13]:

A 3 $$C_{v}=\frac{K_{1}}{\rho }\;\text{and}\;K_{1}=\int_{0}^{l}\frac{A}{c^{2}}\;\text{d}x,$$

where *A* and *c* are evaluated at $\bar{P}$. Furthermore, we can estimate the compliance *C*_t_ of a terminal vessel (figure 3) coupled with a WK3 with proximal resistance, *R*_1_, compliance, *C* and peripheral resistance, *R*_2_ [13]:

A 4 $$C_{t}=\frac{C_{v}R_{2}+C_{v}R_{1}+CR_{2}+C_{v}R_{v}}{R_{2}+R_{1}+R_{v}}.$$

Lumped compliance of terminal vessels coupled to WK_cor_ models with compliances *C*_a_ and *C*_m_ were estimated according to:

A 5 $$C_{t}=C_{v}+C_{a}+C_{m}.$$

The compliance contribution of non-terminal vessels was estimated with *C*_v_ alone. In order to find the total compliance contribution of the vessels distal of a site of truncation, we use the rules for adding capacitors/compliances in series and parallel. The equivalent compliance (*C*_eq,b_) of two daughter vessels in a bifurcation and the equivalent compliance (*C*_eq,a_) of one of the mother vessels and the daughter vessel in an anastomosis is given by (figure 11):

A 6*a* $$C_{\mathrm{eq},b}=C_{d,1}+C_{d,2}$$

and

A 6*b* $$C_{\mathrm{eq},a}=C_{m,1}+\frac{1}{2}C_{d},$$

where *C*_d,1_ and *C*_d,2_ are the lumped compliances of the two daughter vessels in the bifurcation, *C*_d_ is the lumped compliance of the daughter vessel in the anastomosis and *C*_m,1_ is the lumped compliance of one of the mother vessels in the anastomosis. The compliance contribution of the daughter vessel in an anastomosis is thus split equally between the two mothers.

With the lumped compliance, and estimate of total resistance at a site of truncation as described in §2.4.1.1, the distal arteries may be lumped into *WK* models, as illustrated in figure 12.

**A.3.2. Method 2, optimization of lumped parameters**

**A.3.2.1. Parameter sensitivity, correlation and identifiability**

We wanted to assure that the parameters were identifiable, and did so by checking if any of the parameters were highly correlated. The sensitivity of the model output, *y* to the model parameters, *θ* can be calculated by the sensitivity matrix [40]:

A 7 $$S=\frac{\partial y}{\partial \theta }=\left[\begin{array}{ccc} \frac{\partial y}{\partial \theta_{1}(t_{1})} & \cdots & \frac{\partial y}{\partial \theta_{m}(t_{1})}\\ \frac{\partial y}{\partial \theta_{1}(t_{2})} & \cdots & \frac{\partial y}{\partial \theta_{m}(t_{2})}\\ \vdots & \vdots & \vdots \\ \frac{\partial y}{\partial \theta_{1}(t_{n})} & \cdots & \frac{\partial y}{\partial \theta_{m}(t_{n})} \end{array}\right],$$

in which *m* is at most 3, [*θ*_1_, *θ*_2_, *θ*_3_] = [*R*_1_, *C*, *R*_2_], in our case, *y* is the solution of the WK3 ODE, *P*_*WK*3_ and *n* is the number of time points in one period. The sensitivity matrix, *S*, was calculated using forward differences. From the sensitivity matrix, we may calculate the model Hessian ***H*** = ***C***^−1^ = σ^−2^***S***^T^***S***, where σ is the variance and ***C*** is the covariance matrix. The correlation matrix can be calculated as [40]:

A 8 $$c_{i,j}=\frac{C_{i,j}}{\sqrt{C_{i,i}C_{\;j,j}}}.$$

If |*c*_*i*,*j*_| = 1, *i* ≠ *j* then parameters *θ*_*i*_ and *θ*_*j*_ are perfectly correlated. In other words altering *θ*_*i*_ or *θ*_*j*_ has the same effect on *y*, and hence both of them cannot be identified in the same optimization process. In this work, we have treated two parameters as pairwise correlated if |*c*_*i*,*j*_| > 0.86, and with this criterion we found that in most optimization cases either two or more of *R*_1_, *C*, *R*_2_ were pairwise correlated. By keeping *R*_1_ + *R*_2_ constant and only allowing the relative distribution *R*_1_/*R*_2_ to vary, the subset of parameters, ([*θ*_1_, *θ*_2_] = [*R*_1_/*R*_2_, *C*]) was not highly correlated for any of the optimization cases. We therefore used [*R*_1_/*R*_2_, *C*] as the set of parameters to be optimized in Method 2. Furthermore, if the optimum value of *R*_1_ was less than 0, *R*_1_ was set equal to the characteristic impedance, and only *C* was optimized.

**A.4. Computational aspects**

**A.4.1. Creation of reduced networks**

There are approximately 4.7 million unique networks that can be reduced from the 96-artery baseline model shown in figure 1. Solving all of them was infeasible, however, through some initial tests we managed to reduce the number of possible combinations down to approximately 30000. This was done by replacing branches of vessels that had little effect on the pressure and flow waveforms in the arteries of interest (*ε*_Q,avg_ < 0.3 and *ε*_P,avg_ < 0.1).

**A.5. Application to different physiological and pathological states**

As described in §2.6, total arterial resistance and compliance was altered to represent different physiological states. Here, we describe the details on how this was performed. Departing from the parameters obtained from performing network reduction, total arterial compliance, *C*_tot_ was calculated as the sum of compliance contribution of 1D segments, *C*_tot,1D_ and WK3 compliance of terminal vessels, *C*_tot,0D_ according to

A 9 $$C_{\mathrm{tot}}=C_{\mathrm{tot},1D}+C_{\mathrm{tot},0D}=\sum_{k=1}^{N_{v}}C_{v,k}+\sum_{k=1}^{N_{t}}C_{k},$$

where *k* is the summation index, *N*_*v*_ is the number of 1D-segments, with compliance *C*_v,*k*_ (see equation (A 3)) and *N*_*t*_ is the number of terminal vessels with WK3 compliance, *C*_*k*_. As mentioned in §2.6, total arterial resistance was modified by altering the peripheral resistance, *R*_2_ in all outflow WK3 models. However, since part of the resistance contribution is due to resistance in the 1D domain, we used the estimated mean value at the aortic root, ${\bar{P}}_{\mathrm{inlet}}$ (see §2.4.1) as a surrogate measure of the total arterial resistance. Next, we defined a target inlet pressure, ${\bar{P}}_{\mathrm{inlet},\mathrm{target}}$ and updated the peripheral resistance, *R*_2_ in all outflow WK3 models according to the expression

A 10 $$R_{2,k}^{m+1}+R_{1,k}=\frac{{\bar{P}}_{\mathrm{inlet},\mathrm{target}}}{{\left({\bar{P}}_{\mathrm{inlet}}\right)}^{m}}(R_{2,k}^{m}+R_{1,k}),$$

where *k* denotes the relevant outflow segment and *m* is an iteration index. For the case when normal ageing was simulated, ${\bar{P}}_{\mathrm{inlet},\mathrm{target}}$ was set to 110 mmHg (i.e. total arterial resistance was increased with a factor of 1.1 since ${\left({\bar{P}}_{\mathrm{inlet}}\right)}^{0}$ was 100 mmHg). For inflow case 2, it was necessary to increase total arterial resistance to produce physiological pressure waveforms. Here, ${\bar{P}}_{\mathrm{inlet},\mathrm{target}}$ was set to 90 mmHg (i.e. total arterial resistance was increased by a factor of 1.67 since ${\left({\bar{P}}_{\mathrm{inlet}}\right)}^{0}$ was 54 mmHg). Four iterations were sufficient to reach ${\bar{P}}_{\mathrm{inlet},\mathrm{target}}$. In order to decrease total arterial compliance by a factor of 2, we defined a target compliance *C*_tot, target_ = *C*_tot_/2, and increased the stiffness parameter of proximal arteries by a factor of 2.5 and all others by a factor of 1.5. The following segment Ids were considered as proximal segments; 1, 2, 3, 4, 5, 14, 15, 18, 19, 27, 28 (see the electronic supplementary material). Next, we estimated the compliance contribution of 1D segments after this modification, *C*_tot,1D,mod_, and calculated the target WK3 compliance, *C*_tot,0D,target_ according to

A 11 $$C_{\mathrm{tot},0D,\mathrm{target}}=C_{\mathrm{tot},\mathrm{target}}-C_{\mathrm{tot},1D,\mathrm{mod}}.$$

Finally, we updated the individual WK3 compliances according to

A 12 $$C_{k,\mathrm{mod}}=C_{k}\frac{C_{\mathrm{tot},0D,\mathrm{target}}}{C_{\mathrm{tot},0D}},$$

where *C*_*k*,mod_ is the modified WK3 compliance for terminal segment *k*.

## Appendix B. Results

**B.1. Framework for optimizing topological complexity**

In order to ensure that interaction between different regions in the network and that pressure propagation was correctly captured throughout the larger systemic arteries, a threshold based on pressure waveforms at four locations was used in figure 13. Here, the average *ε*_*P*,avg_ for the aortic root, right common carotid, right brachial and left femoral artery pressure waveforms was required to be less than 0.4%.

**B.2. Application to different physiological and pathological states**

Figures 14–17 show the results from the second part of our study, where we re-parametrized a series of optimal networks to represent (1) normal ageing, (2) a pathological state of aortic coarctation and (3) states of different heart rate, ejection time and stroke volume, as described in §2.6. Corresponding error metrics are given in table 1.
