# Supplementary material for: Optimization of topological complexity for one-dimensional arterial blood flow models
Source: J R Soc Interface. 2018 Dec 12;15(149):20180546. doi: 10.1098/rsif.2018.0546 (PMC6303799; doi:10.1098/rsif.2018.0546)
Supplement: Supplementary material for optimization of complexity for arterial blood flow models [file rsif20180546supp1.pdf]

Optimization of topological complexity for 1D arterial blood flow models

Fredrik E. Fossan, Jorge Mariscal-Harana, Jordi Alastruey, Leif R. Hellevik

Supplementary material 1

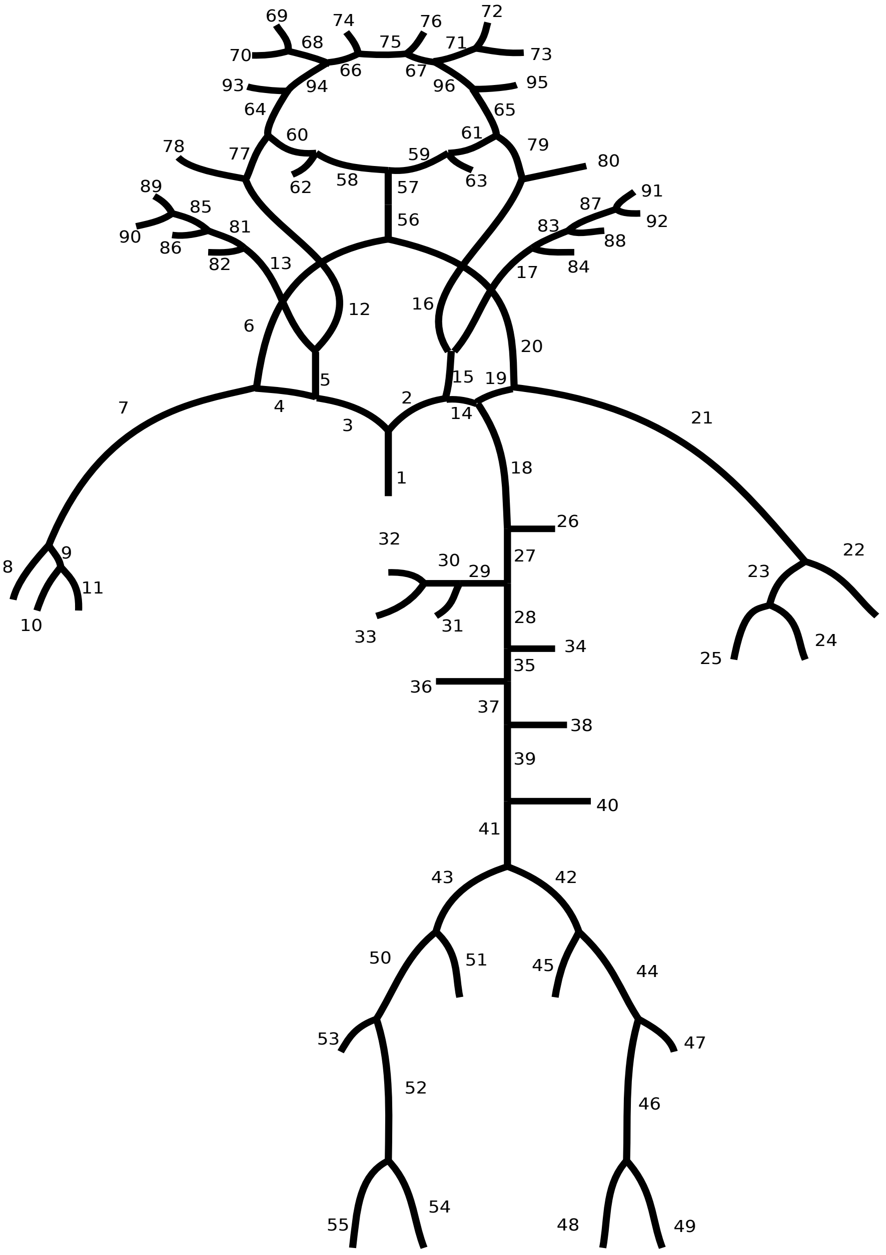

Figure 1: Visualization of the 96-artery model with segment Ids corresponding to Tables 1- 2

Table 1: Model parameters for the 96-artery baseline model, including length of segment, inlet and outlet radius (R_{in} and R_{out}), inlet and outlet wavespeed (c_{in} and c_{out}) and WK3 model parameters (R_1, R_2 and C).

Id	Name	Length $[cm]$	$R_{in} \rightarrow R_{out}$ $[mm]$	$c_{in} \rightarrow c_{out}$ $[m/s]$	R_1 $[Pa\,s\,m^{-3}]$ $\cdot 10^{-9}$	R_2 $[Pa\,s\,m^{-3}]$ $\cdot 10^{-9}$	C $[m^3\,Pa^{-1}]$ $\cdot 10^{10}$
1	Ascending Aorta	6.0	$14.87 \rightarrow 14.7$	$4.6 \rightarrow 4.6$			
2	Aortic Arch I	2.0	$12.9 \rightarrow 12.9$	$4.6 \rightarrow 4.6$			
3	Brachiocephalic Artery	3.4	$6.99 \rightarrow 6.99$	$4.64 \rightarrow 4.64$			
4	Right Subclavian Artery I	3.4	$6.0 \rightarrow 5.5$	$4.7 \rightarrow 4.75$			
5	Right Carotid Artery	9.4	$4.0 \rightarrow 3.45$	$5.13 \rightarrow 5.45$			
6	Right Vertebral Artery	14.9	$1.85 \rightarrow 1.4$	$7.54 \rightarrow 8.64$			
7	Right Brachial Artery	42.2	$5.5 \rightarrow 2.36$	$4.75 \rightarrow 6.61$			
8	Right Radial Artery	23.5	$1.3 \rightarrow 1.07$	$8.93 \rightarrow 9.66$	2.825	5.324	0.529
9	Right Ulnar Artery I	6.7	$2.15 \rightarrow 2.15$	$6.96 \rightarrow 6.96$			
10	Right Interosseous Artery	7.9	$0.91 \rightarrow 0.91$	$10.22 \rightarrow 10.22$	4.163	7.316	0.38
11	Right Ulnar Artery II	17.1	$2.0 \rightarrow 1.83$	$7.24 \rightarrow 7.59$	0.764	1.911	1.538
12	Right Internal Carotid Artery	17.8	$2.85 \rightarrow 2.15$	$5.98 \rightarrow 6.96$			
13	Right External Carotid Artery I	4.1	$2.5 \rightarrow 2.25$	$6.41 \rightarrow 6.79$			
14	Aortic Arch II	3.9	$12.4 \rightarrow 12.4$	$4.6 \rightarrow 4.6$			
15	Left Carotid Artery	13.9	$3.7 \rightarrow 3.22$	$5.29 \rightarrow 5.62$			
16	Left Internal Carotid Artery	17.8	$2.65 \rightarrow 2.05$	$6.21 \rightarrow 7.14$			
17	Left External Carotid Artery I	4.1	$2.35 \rightarrow 2.15$	$6.63 \rightarrow 6.96$			
18	Descending Thoracic Aorta I	5.2	$11.0 \rightarrow 9.99$	$4.6 \rightarrow 4.61$			
19	Left Subclavian Artery I	3.4	$5.5 \rightarrow 5.0$	$4.75 \rightarrow 4.83$			
20	Left Vertebral Artery	14.8	$1.85 \rightarrow 1.4$	$7.54 \rightarrow 8.64$			
21	Left Brachial Artery	42.2	$4.65 \rightarrow 2.36$	$4.91 \rightarrow 6.61$			
22	Left Radial Artery	23.5	$1.3 \rightarrow 1.07$	$8.93 \rightarrow 9.66$	2.825	5.324	0.529
23	Left Ulnar Artery I	6.7	$2.15 \rightarrow 2.15$	$6.96 \rightarrow 6.96$			
24	Left Interosseous Artery	7.9	$0.91 \rightarrow 0.91$	$10.22 \rightarrow 10.22$	4.163	7.316	0.38
25	Left Ulnar Artery II	17.1	$2.0 \rightarrow 1.83$	$7.24 \rightarrow 7.59$	0.764	1.911	1.538
26	Intercostal Artery	8.0	$1.2 \rightarrow 1.2$	$9.23 \rightarrow 9.23$	2.164	0.06	3.811
27	Descending Thoracic Aorta II	10.4	$8.7 \rightarrow 7.1$	$4.61 \rightarrow 4.64$			
28	Abdominal Aorta I	5.3	$6.1 \rightarrow 6.1$	$4.69 \rightarrow 4.69$			
29	Celiac Artery	2.0	$3.8 \rightarrow 3.8$	$5.23 \rightarrow 5.23$			
30	Thirty	1.0	$3.0 \rightarrow 3.0$	$5.82 \rightarrow 5.82$			
31	Hepatic Artery	7.1	$2.9 \rightarrow 2.75$	$5.92 \rightarrow 6.09$	0.272	4.341	0.763
32	Splenic Artery	6.6	$2.4 \rightarrow 2.4$	$6.55 \rightarrow 6.55$	0.384	1.599	1.955
33	Gastric Artery	6.3	$2.0 \rightarrow 2.0$	$7.24 \rightarrow 7.24$	0.611	2.053	1.482
34	Superior Mensenteric Artery	5.9	$3.0 \rightarrow 3.0$	$5.82 \rightarrow 5.82$	0.218	1.011	3.096
35	Abdominal Aorta II	1.0	$5.9 \rightarrow 5.9$	$4.7 \rightarrow 4.7$			
36	Left Renal Artery	3.2	$2.6 \rightarrow 2.6$	$6.27 \rightarrow 6.27$	0.313	0.568	4.93
37	Abdominal Aorta III	1.0	$5.7 \rightarrow 5.7$	$4.72 \rightarrow 4.72$			
38	Right Renal Artery	3.2	$2.6 \rightarrow 2.6$	$6.27 \rightarrow 6.27$	0.313	0.568	4.93
39	Abdominal Aorta IV	10.6	$5.5 \rightarrow 5.3$	$4.75 \rightarrow 4.77$			
40	Inferior Mesenteric Artery	5.0	$1.6 \rightarrow 1.6$	$8.12 \rightarrow 8.12$	1.07	3.44	0.881
41	Abdominal Aorta V	1.0	$5.2 \rightarrow 5.0$	$4.79 \rightarrow 4.83$			
42	Left Common Iliac Artery	5.8	$3.68 \rightarrow 3.5$	$5.3 \rightarrow 5.41$			
43	Right Common Iliac Artery	5.9	$3.68 \rightarrow 3.5$	$5.3 \rightarrow 5.41$			
44	Left External Iliac Artery	14.4	$2.8 \rightarrow 2.7$	$6.03 \rightarrow 6.15$			
45	Left Internal Iliac Artery	5.0	$2.63 \rightarrow 2.63$	$6.24 \rightarrow 6.24$	0.303	4.177	0.79
46	Left Femoral Artery	44.3	$2.5 \rightarrow 1.9$	$6.41 \rightarrow 7.44$			
47	Left Deep Femoral Artery	12.6	$1.5 \rightarrow 1.25$	$8.37 \rightarrow 9.08$	1.972	1.898	1.278
48	Left Posterior Tibial Artery	32.1	$1.55 \rightarrow 1.4$	$8.24 \rightarrow 8.64$	1.488	1.47	1.662
49	Left Anterior Tibial Artery	34.3	$1.3 \rightarrow 1.15$	$8.93 \rightarrow 9.39$	2.396	2.105	1.121
50	Right External Iliac Artery	14.5	$2.8 \rightarrow 2.7$	$6.03 \rightarrow 6.15$			
51	Right Internal Iliac Artery	5.0	$2.63 \rightarrow 2.63$	$6.24 \rightarrow 6.24$	0.303	4.177	0.79
52	Right Femoral Artery	44.4	$2.5 \rightarrow 1.9$	$6.41 \rightarrow 7.44$			
53	Right Deep Femoral Artery	12.7	$1.5 \rightarrow 1.25$	$8.37 \rightarrow 9.08$	1.972	1.898	1.278
54	Right Posterior Tibial Artery	32.2	$1.55 \rightarrow 1.4$	$8.24 \rightarrow 8.64$	1.488	1.465	1.666
55	Right Anterior Tibial Artery	34.4	$1.3 \rightarrow 1.15$	$8.93 \rightarrow 9.39$	2.396	2.097	1.124

Table 2: Model parameters for the 96-artery baseline model, including length of segment, inlet and outlet radius (R_{in} and R_{out}), inlet and outlet wavespeed (c_{in} and c_{out}) and WK3 model parameters (R_1, R_2 and C).

Id	Name	Length $[cm]$	$R_{in} \rightarrow R_{out}$ $[mm]$	$c_{in} \rightarrow c_{out}$ $[m/s]$	R_1 $[Pa\,s\,m^{-3}]$ $\cdot 10^{-9}$	R_2 $[Pa\,s\,m^{-3}]$ $\cdot 10^{-9}$	C $[m^3\,Pa^{-1}]$ $\cdot 10^{10}$
56	Left Posterior Communicating Artery	1.5	2.0 → 1.9	7.24 → 7.44			
57	Basilar Artery I	1.0	1.9 → 1.35	7.44 → 8.79			
58	Right Posterior Cerebral Artery I	0.4	0.95 → 0.95	10.07 → 10.07			
59	Left Posterior Cerebral Artery I	0.4	0.95 → 0.95	10.07 → 10.07			
60	Right Posterior Communicating Artery	0.4	0.75 → 0.75	10.83 → 10.83			
61	Left Posterior Communicating Artery	0.4	0.75 → 0.75	10.83 → 10.83			
62	Right Posterior Cerebral Artery II	5.9	1.0 → 0.9	9.89 → 10.25	4.271	3.903	0.167
63	Left Posterior Cerebral Artery II	5.9	1.0 → 0.9	9.89 → 10.25	4.271	3.903	0.167
64	Right Distal Internal Carotid Artery I	0.4	1.95 → 1.9	7.34 → 7.44			
65	Left Distal Internal Carotid Artery I	0.4	1.95 → 1.9	7.34 → 7.44			
66	Right Anterior Cerebral Artery I	1.2	1.05 → 1.0	9.72 → 9.89			
67	Left Anterior Cerebral Artery I	1.2	1.05 → 1.0	9.72 → 9.89			
68	Right Middle Cerebral Artery (M1)	0.8	1.5 → 1.4	8.37 → 8.64			
69	Right Superior Middle Cerebral Artery (M2)	7.1	1.0 → 1.0	9.89 → 9.89	3.339	3.208	0.206
70	Right Inferior Middle Cerebral Artery (M2)	7.0	1.0 → 1.0	9.89 → 9.89	3.339	3.208	0.206
71	Left Middle Cerebral Artery (M1)	0.8	1.5 → 1.4	8.37 → 8.64			
72	Left Superior Middle Cerebral Artery (M2)	7.1	1.0 → 1.0	9.89 → 9.89	3.339	3.208	0.206
73	Left Inferior Middle Cerebral Artery (M2)	7.0	1.0 → 1.0	9.89 → 9.89	3.339	3.208	0.206
74	Right Anterior Cerebral Artery II	2.4	0.9 → 0.85	10.25 → 10.44	4.875	4.342	0.149
75	Anterior Communicating Artery	0.4	0.65 → 0.65	11.24 → 11.24			
76	Left Anterior Cerebral Artery II	2.4	0.9 → 0.85	10.25 → 10.44	4.875	4.342	0.149
77	Right Internal Carotid Sinus	1.1	2.15 → 1.95	6.96 → 7.34			
78	Right Opthalmic Artery	1.1	0.5 → 0.25	11.9 → 13.11	70.772	114.372	0.024
79	Left Internal Carotid Sinus	1.1	2.15 → 1.95	6.96 → 7.34			
80	Left Opthalmic Artery	1.1	0.5 → 0.25	11.9 → 13.11	70.772	114.372	0.024
81	Right External Carotid Artery II	6.1	2.0 → 1.75	7.24 → 7.76			
82	Right Superior Thyroid	10.1	1.4 → 1.4	8.64 → 8.64	1.488	3.942	0.751
83	Left External Carotid Artery II	6.1	2.0 → 1.75	7.24 → 7.76			
84	Left Superior Thyroid	10.1	1.1 → 0.8	9.56 → 10.63	5.605	11.67	0.245
85	Right Superior Temporal Artery	6.1	1.6 → 1.5	8.12 → 8.37			
86	Right Maxillary Artery	9.1	1.1 → 0.5	9.56 → 11.9	16.055	29.221	0.096
87	Left Superior Temporal Artery	6.1	1.6 → 1.5	8.12 → 8.37			
88	Left Maxillary Artery	9.1	1.1 → 0.5	9.56 → 11.9	16.055	29.221	0.096
89	Right Superior Temporal Frontal Artery	10.0	1.1 → 0.7	9.56 → 11.03	7.596	15.137	0.188
90	Right Superior Temporal Parietal Artery	10.1	1.1 → 0.7	9.56 → 11.03	7.596	15.137	0.188
91	Left Superior Temporal Frontal Artery	10.0	1.1 → 0.7	9.56 → 11.03	7.596	15.137	0.188
92	Left Superior Temporal Parietal Artery	10.1	1.1 → 0.7	9.56 → 11.03	7.596	15.137	0.188
93	Right Anterior Choroidal Artery	3.6	0.75 → 0.65	10.83 → 11.24	8.975	7.181	0.087
94	Right Distal Internal Carotid Artery II	0.4	1.9 → 1.9	7.44 → 7.44			
95	Left Anterior Choroidal Artery	3.6	0.75 → 0.65	10.83 → 11.24	8.975	7.181	0.087
96	Left Distal Internal Carotid Artery II	0.4	1.9 → 1.9	7.44 → 7.44			
